# Endoscopic Assessment Prior to Bariatric Surgery in Saudi Arabia

**DOI:** 10.7759/cureus.36157

**Published:** 2023-03-14

**Authors:** Mahdi E Aljaroudi, Murtaga Makki, Mohammed Almulaify, Abdullah Alshabib, Hind Alfaddagh, Hassan Alzahrani, Sulaiman Alghamdi, Wael Alsualiman, Jaafar Alsalman, Mousa J Alhaddad

**Affiliations:** 1 Gastroenterology and Hepatology, Dammam Medical Complex, Dammam, SAU; 2 Internal Medicine, Dammam Medical Complex, Dammam, SAU

**Keywords:** helicobacter pylori, esophagogastroduodenoscopy, preoperative evaluation, bariatric surgery, obesity

## Abstract

Background: There are marked local inconsistencies in the Arabian Peninsula about the role of preoperative esophagogastroduodenoscopy (EGD) in bariatric surgery. Thus, this study was conducted to determine the frequency of endoscopic and histological findings in the Saudi population presenting for pre-bariatric surgery evaluation.

Material and Methods: This was a retrospective study that included all the patients who were evaluated by EGD at Dammam Medical Complex, Dammam, Saudi Arabia, between 2018 and 2021 as a part of their pre-bariatric-surgery evaluation.

Results: A total of 684 patients were included. They consisted of 250 male and 434 female patients (36.5% and 63.5%, respectively). The mean ± standard deviation for the patients' age and body mass index (BMI) were 36.4±10.6 years and 44.6±5.1 kg/m^2^, respectively. Significant endoscopic or histopathological findings as defined by the presence of large (≥ 2 cm) hiatus hernia, esophagitis, gastroesophageal reflux disease (GERD), Barrett esophagus, gastric ulcer, duodenal ulcer, or intestinal metaplasia were found in 143 patients (20.9%); 364 patients (53.2%) were diagnosed to have *Helicobacter pylori* infection.

Conclusion: The high number of significant endoscopic and histopathological findings in our study supports the routine use of preoperative EGD in all bariatric surgery patients. However, omitting EGD before Roux-en-Y gastric bypass (RYGB) in asymptomatic patients is still reasonable as the most frequently found significant findings, esophagitis, and hiatus hernia, are less likely to impact the operative plans in RYGB. Similarly, active surveillance and treatment of *H. pylori *infections in obese patients are important but it is not clear whether *H. pylori *eradication should be done before bariatric surgery.

## Introduction

Obesity represents a major global problem and a leading cause of death in the world accounting for around 3.4 million deaths annually. It is estimated that 20% of the world's adult population will be obese by the year 2030 [1]. In Saudi Arabia, the weighted prevalence of obesity is estimated to be between 24.7% and 35.5%, which is higher than the global average [2-4]. The impact of overweight and obesity in Saudi Arabia is found to directly cost a total of $3.8 billion annually, which was equal to 4.3% of the total health expenditure in the country in 2019 [5].

Bariatric surgery results in a greater and more durable weight loss than the best available nonsurgical interventions for obesity, regardless of the type of bariatric procedures used [6]. Beyond weight loss, the effect of bariatric surgery extends to enhancing glycemic control [7,8], improving lipid profile [9], controlling elevated blood pressure [10], achieving type 2 diabetes mellitus remission and metabolic syndrome resolution [11,12], alleviating obstructive sleep apnea [13], augmenting renal functions [14], treating nonalcoholic fatty liver disease [15], improving quality of life and body image [16], improving fertility and sexual health [17,18], lowering cardiovascular events [19], decreasing cancer risks [20], prolonging long-term survival [19], and decreasing medical cost [21].

With the knowledge of these effects, the rate of bariatric surgeries continued to increase over time with more than 500,000 operations performed annually in the world [22]. Bariatric surgeries are also becoming more popular in the Saudi community. Around 52% of the Saudi population think that surgery is the best intervention to cure obesity [23], and 53% of 1129 interviewed Saudi individuals stated that they would seek a bariatric surgeon's help if they were morbidly obese [24], and more than 25,000 procedures are performed every year by surgeons in Saudi Arabia [25].

Esophagogastroduodenoscopy (EGD) assessment before bariatric operations differs in its necessity. The European Association for Endoscopic Surgery provided a conditional recommendation for routine preoperative EGD [26]. Similarly, the Saudi Arabian Society for Metabolic and Bariatric Surgery advises EGD as a routine preoperative investigation for all bariatric surgeries in its 2020-2021 guidelines update [25]. Others, on the other hand, conclude that standard preoperative assessment by EGD is not indicated in patients who are planned for bariatric surgery as the number needed to screen to find clinically significant abnormalities is high and recommend EGD only in patients with upper gastrointestinal symptoms [27-29].

Owing to this non-consensus regarding the need for routine preoperative EGD in bariatric surgery patients, this study was conducted to determine the frequency of endoscopic and histopathologic findings in the Saudi population presenting for pre-bariatric surgery evaluation.

## Materials and methods

This was a retrospective study that included all the patients who were evaluated by EGD at Dammam Medical Complex, Dammam, Saudi Arabia, between 2018 and 2021 as a part of their pre-bariatric-surgery evaluation. Patients who had a previous gastric surgery that might alter the normal anatomy and histology (e.g., previous bariatric surgery) were excluded.

The data about the patients' age, gender, comorbid conditions, body mass index, use of antiplatelets and proton pump inhibitors, laboratory investigations, endoscopic results, and histopathological findings were collected. Significant endoscopic or histopathological findings were defined as the presence of large (≥ 2 cm) hiatus hernia, esophagitis, gastroesophageal reflux disease (GERD), Barrett's esophagus, gastric ulcer, duodenal ulcer, or intestinal metaplasia.

The data were analyzed using the Python programming language version 3.7.6 (Python Software Foundation, Wilmington, Delaware, United States) with the use of the SciPy library 1.4.1 (Enthought, Inc., Austin, Texas, United States) and Statsmodels module (v0.11.1). Descriptive statistics (i.e., mean, standard deviation, count, and percentage) were calculated as necessary. Categorical variables were compared with the Chi-square test and continuous variables were compared with the two-sample t-test. A p-value of less than 0.05 was assumed to indicate statistical significance.

The research project was approved and monitored by Dammam Medical Complex Institutional Review Board (Log Number: 27, Protocol Number: END-01, dated October 30, 2022), and all data were used only for research purposes.

## Results

**Figure 1:**
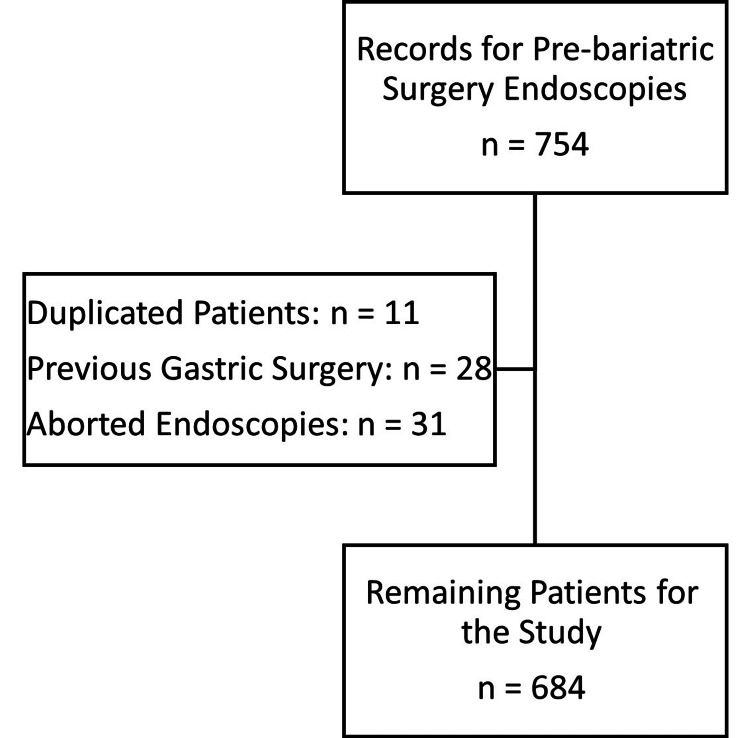
Flowchart for the reviewed patients

A total of 754 records for pre-bariatric surgery endoscopy were retrieved in the study period. Eleven records were found to be duplicates and 28 patients had a history of previous gastric surgery and were excluded. Thirty-one patients did not tolerate the endoscopic procedures and were also excluded. The remaining 684 patients were included in the analysis (Figure 1).

**Figure 2:**
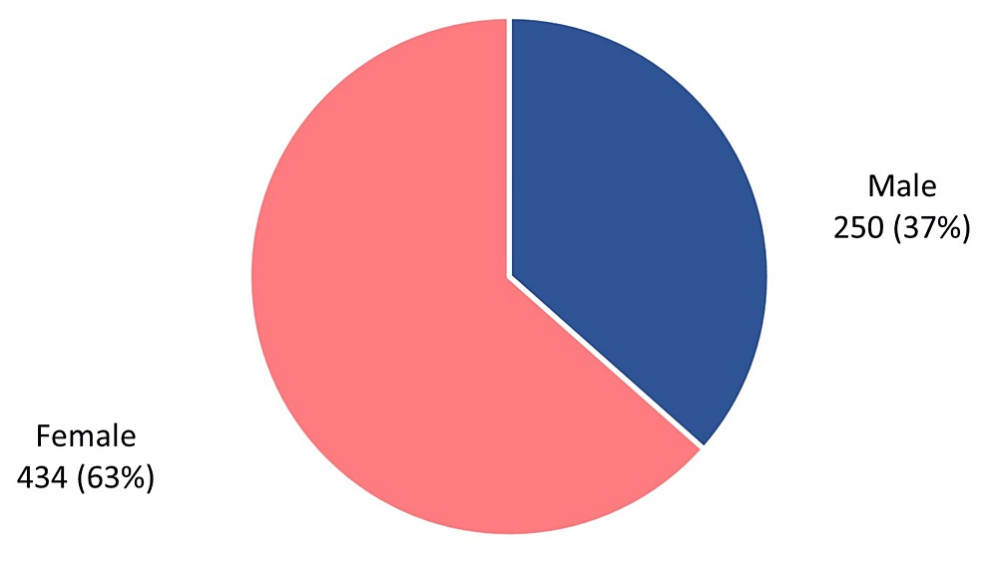
Patients' sex (n = 684)

The included patients consisted of 250 male and 434 female patients (36.5% and 63.5%, respectively) with a male-to-female ratio of 0.58 (Figure 2).

**Figure 3:**
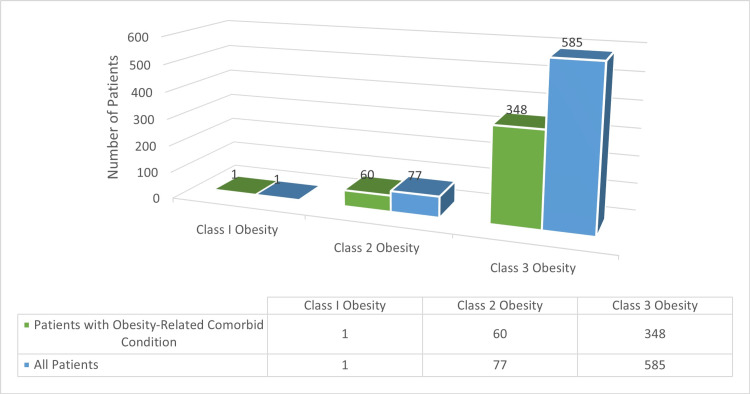
Patients' obesity class (n = 633) Most of the studied patients were of the third World Health Organization (WHO) obesity class as defined by a body mass index (BMI) of ≥40 kg/m^2^ (585 patients, 88.2%). Most of them also had at least one obesity-related comorbid condition (409 patients, 61.2%).  Examples of these conditions include type 2 diabetes mellitus, hypertension, hyperlipidemia, nonalcoholic fatty liver disease, gastroesophageal reflux disease, debilitating osteoarthritis, and obstructive sleep apnea. The BMI was not recorded for 21 patients.

Most of the patients were Saudi (673 patients, 98.4%). The mean ± standard deviation for the patients' age was 36.4±10.6 years. One hundred and seventy-five patients (25.6%) were hypertensive and 166 patients (24.3%) were diabetic. The mean ± standard deviation for BMI was 44.6±5.1 kg/m^2^ (Figure 3).

Forty-two patients (6.1%) were known to have a history of gastroesophageal reflux disease (GERD, 136 patients (19.9%) were using a proton pump inhibitor (PPI), 21 patients (3.1%) were on aspirin as a single antiplatelet therapy while three patients (0.4%) were on dual antiplatelets. Refer to Table 1 for the patients' demographics and medical backgrounds.

**Table 1:** Patients' demographics and medical backgrounds (n = 684)

Characteristic		n (%)
Age (Mean ± SD, years)		36.44±10.61
Gender	Male	250 (36.55%)
	Female	434 (63.45%)
Nationality	Saudi	673 (98.39%)
	Non-Saudi	11 (1.61%)
BMI (Mean ± SD)		44.6±5.1
Hypertension		175 (25.58%)
Diabetes Miletus		166 (24.27%)
Gastroesophageal Reflux Disease (GERD)		42 (6.14%)
Ischemic Heart Disease		12 (1.75%)
Non-Alcoholic Fatty Liver Disease (NAFLD)		11 (1.61%)
Chronic Kidney Disease		6 (0.88%)
Heart Failure		4 (0.58%)
Using Proton Pump Inhibitors (PPIs)		136 (19.88%)
Using Non-Steroidal Anti-Inflammatory Drugs (NSAIDs)		59 (8.63%)
Using Antiplatelets	Aspirin only	21 (3.07%)
	Dual antiplatelets	3 (0.44%)

One hundred and eighty-three patients (26.8%) had a hemoglobin of less than 12 g/dl. Among those anemic patients, the mean ± standard deviation for the patients' mean corpuscular volume (MCV) was 74.2 ± 10.2 fl. Thrombocytopenia and thrombocytosis were found in 10 (1.5%) and 31 (4.5%) patients, respectively. Thirteen patients (1.9%) had a creatinine level of more than 1.2 mg/dl; 187 (27.3%) and 134 (19.6%) patients were found to have high low-density lipoprotein (LDL) cholesterol (>130 mg/dl) and low high-density lipoprotein (HDL) cholesterol (<40 mg/dl), respectively. Hypertriglyceridemia (>150 mg/dl) was present in 131 patients (19.2%). Refer to Table 2 for the patients' laboratory results.

**Table 2:** Patients' laboratory results (n = 684)

Characteristic	Mean ± SD	Normal Range
White Blood Cells (WBCs)	7.23±2.99	4-10 ×10^9^/l
Hemoglobin	12.92±1.86	11.5-15.5 g/dl
Mean Corpuscular Volume (MCV)	79.89±8.87	80-100 fl
Platelet Count	310.36±83.76	150-450 ×10^9^/l
Sodium (Na)	138.95±5.7	136-145 mmol/l
Potassium (K)	4.18±0.4	3.5-5.1 mmol/l
Creatinine	0.73±0.21	0.5-0.9 mg/dl
Urea	24.41±8.82	0-50 mg/dl
Total Bilirubin	0.54±0.33	0-1 mg/dl
Direct Bilirubin	0.15±0.12	0-0.2 mg/dl
Alanine Aminotransferase (ALT)	26.92±17.91	0-33 u/l
Aspartate Aminotransferase (AST)	21.64±10.63	0-32 u/l
Alkaline Phosphatase (ALP)	78.89±23.94	35-104 u/l
Gamma-Glutamyl Transferase (GGT)	36.05±39.27	0-38 u/l
Albumin	4.15±3.87	3.2-4.8 g/dl
Total Cholesterol	187.28±38.68	0-200 mg/dl
Low-Density Lipoprotein (LDL) Cholesterol	120.38±35.51	100-130 mg/dl
High-Density Lipoprotein (HDL) Cholesterol	47.17±12.71	40-60 mg/dl
Triglycerides	119.15±64.47	0-150 mg/dl
Prothrombin Time (PT)	11.93±1.78	13-17 s
Partial Thromboplastin Time (PTT)	31.31±6.14	25-35 s
International Normalized Ratio (INR)	1.0±1.47	0.9-1.2

**Figure 4:**
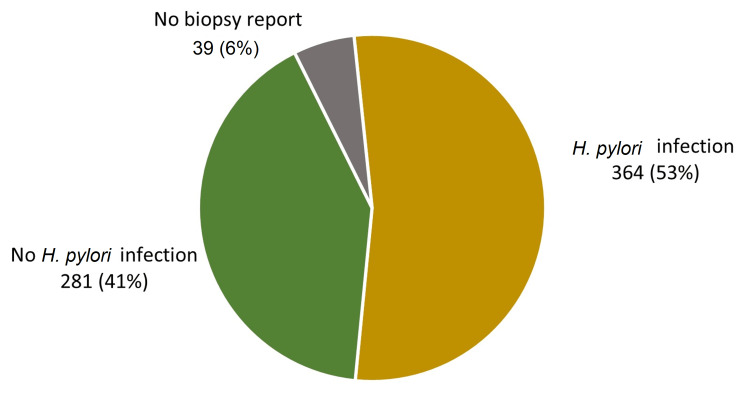
Helicobacter pylori infection (n = 684) Three hundred and sixty-four patients (53%) were diagnosed to have *H. pylori *infections based on a histopathological assessment of their biopsies. No biopsy report was found in 39 patients (6%). H. pylori: *Helicobacter pylori*

Simple gastritis was the most common endoscopic finding appearing in 351 patients (51.3%) followed by hiatus hernia (206 patients, 30.1%), simple duodenitis (89 patients, 13.0%), and esophagitis (75 patients, 11.0%). Gastric and duodenal ulcers were found in 17 (2.5%) and 10 (1.5%) patients, respectively. No biopsy report was found in 39 patients. Three hundred and sixty-four patients (53.2%) were diagnosed to have *H. pylori *infections based on a histopathological assessment of their biopsies (Figure 4). None of the study patients had malignant lesions. Refer to Table 3 for the patients' endoscopic and histopathological findings.

**Table 3:** Patients' endoscopic and histopathological findings (n = 684)

Characteristic		n(%)
Hiatus Hernia	Small (< 2 cm)	171 (25.0%)
	Large (≥ 2 cm)	35 (5.12%)
Esophagitis		75 (10.96%)
Barrett's Esophagus		1 (0.15%)
Simple Gastritis		351 (51.32%)
Gastric Ulcers		17 (2.49%)
Simple Duodenitis		89 (13.01%)
Duodenal Ulcers		10 (1.46%)
Benign Polyps		12 (1.75%)
Intestinal Metaplasia		12 (1.75%)
Helicobacter pylori		364 (53.22%)

**Figure 5:**
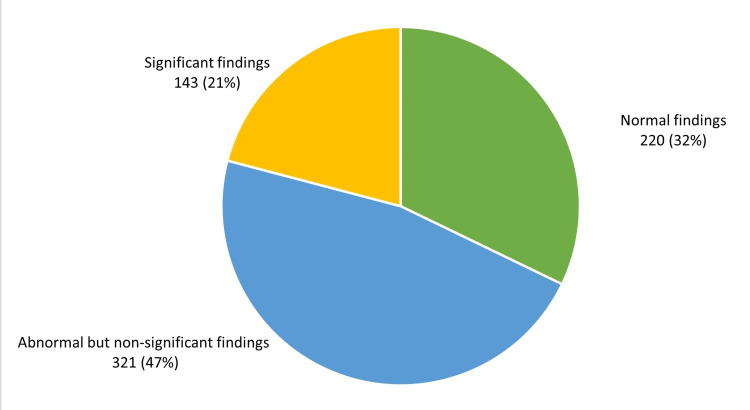
Patients' endoscopic and histological findings (n= 684) Significant endoscopic or histopathological findings (excluding H. pylori infections) as defined by the presence of large (≥ 2 cm) hiatus hernia, esophagitis, GERD, Barrett's esophagus, gastric ulcer, duodenal ulcer, or intestinal metaplasia were found in 143 patients (21%).

Significant endoscopic or histopathological findings as defined by the presence of large (≥ 2 cm) hiatus hernia, esophagitis, GERD, Barrett's esophagus, gastric ulcer, duodenal ulcer, or intestinal metaplasia were found in 143 patients (20.9%) (Figure 5). 

There were no statistically significant differences between the patients with significant findings and the patients without them in age, BMI, diabetes mellitus, hypertension, and use of non-steroidal anti-inflammatory drugs (NSAIDs) or antiplatelets but the patients with no significant findings had higher percentages of female patients (66.2% vs 53.2%) and of using PPIs (21.6% vs 13.3%) than the patients with significant findings (p-values = 0.005 and 0.035, respectively). Patients with significant findings had higher international normalized ratio (INR) values than the patients with no significant findings (1.3±3.3 vs 0.9±0.1, p-value = 0.038). Refer to Table 4 for the differences between patients with significant endoscopic and histopathological findings and patients without significant findings.

**Table 4:** Comparison between patients with significant endoscopic and histopathological findings^ and patients without significant findings (n = 684) ^ Significant findings were defined as having large (≥ 2 cm) hiatus hernia, esophagitis, GERD, Barrett esophagus, gastric ulcer, duodenal ulcer, or intestinal metaplasia. * Significant at p-value of less than 0.05 ALT: alanine aminotransferase; ALP: alkaline phosphatase, AST: aspartate aminotransferase, BMI: body mass index, GGT: gamma-glutamyl transferase, GERD: gastroesophageal reflux disease, INR: international normalized ratio, NAFLD: non-alcoholic fatty liver disease, NSAIDs: non-steroidal anti-inflammatory drugs, PT: prothrombin time, PTT: partial thromboplastin time, PPI: proton pump inhibitor, WBC: white blood count

Characteristic	No Significant Findings (n = 541)	Significant Findings (n = 143)	P value
Age, mean ± SD	36.49±10.53	36.24±10.94	0.802
Female, count (%)	358 (66.17%)	76 (53.15%)	0.005*
Non-Saudi, count (%)	10 (1.85%)	1 (0.7%)	0.550
BMI, mean ± SD	44.69±5.06	44.25±5.23	0.367
Diabetes Miletus, count (%)	136 (25.14%)	30 (20.98%)	0.356
Hypertension, count (%)	140 (25.88%)	35 (24.48%)	0.815
GERD, count (%)	32 (5.91%)	10 (6.99%)	0.778
NAFLD count (%)	7 (1.29%)	4 (2.8%)	0.370
Ischemic Heart Disease, count (%)	10 (1.85%)	2 (1.4%)	0.995
Using NSAIDs, count (%)	49 (9.06%)	10 (6.99%)	0.539
Using Antiplatelet Therapy, count (%)	19 (3.51%)	5 (3.5%)	0.805
Using PPIs, count (%)	117 (21.63%)	19 (13.29%)	0.035*
WBC, mean ± SD, ×10^9^/l	7.11±2.71	7.69±3.91	0.052
Hemoglobin, mean ± SD, g/dl	12.88±1.87	13.09±1.85	0.270
Platelet Count, mean ± SD, ×10^9^/l	310.59±83.26	309.43±86.07	0.890
Creatinine, mean ± SD, mg/dl	0.73±0.21	0.72±0.23	0.691
Total Bilirubin, mean ± SD, mg/dl	0.53±0.31	0.57±0.39	0.209
ALT, mean ± SD, u/l	26.26±17.1	29.57±20.72	0.065
AST, mean ± SD, u/l	21.23±10.15	23.32±12.29	0.051
ALP, mean ± SD, u/l	78.38±23.39	80.75±25.92	0.442
GGT, mean ± SD, u/l	35.3±35.61	39.14±51.74	0.347
Albumin, mean ± SD, g/dl	4.2±4.32	3.94±0.35	0.515
PT, mean ± SD, s	11.89±1.39	12.11±2.9	0.250
PTT, mean ± SD, s	31.46±6.32	30.71±5.33	0.242
INR, mean ± SD	0.94±0.09	1.25±3.33	0.038*

There were no statistically significant differences between the patients with and without *Helicobacter pylori *infections in age, gender, BMI, diabetes mellitus, hypertension, and use of NSAIDs or antiplatelets. Refer to Table 5 for the differences between the patients with and without *H. Pylori *Infection.

**Table 5:** Comparison between patients with and without H. pylori infection (n = 645) * Significant at p-value of less than 0.05 ALT: alanine aminotransferase, ALP: alkaline phosphatase, AST: aspartate aminotransferase, BMI: body mass index, GGT: gamma-glutamyl transferase, GERD: gastroesophageal reflux disease, INR: international normalized ratio, NAFLD: non-alcoholic fatty liver disease, NSAIDs: non-steroidal anti-inflammatory drugs, PT: prothrombin time, PTT: partial thromboplastin time, PPI: proton pump inhibitor, WBC: white blood count; *H. pylori*: *Helicobacter pylori*

Characteristic	*H. Pylori* (n = 364)	No *H. Pylori* (n = 281)	P-value
Age, mean ± SD	37.15±10.26	35.68±10.96	0.081
Female, count (%)	219 (60.16%)	188 (66.9%)	0.094
Non-Saudi, count (%)	2 (0.55%)	9 (3.2%)	0.023*
BMI, mean ± SD	44.73±5.12	44.18±4.97	0.175
Diabetes Miletus, count (%)	90 (24.73%)	66 (23.49%)	0.786
Hypertension, count (%)	98 (26.92%)	67 (23.84%)	0.425
GERD, count (%)	21 (5.77%)	17 (6.05%)	0.985
NAFLD count (%)	7 (1.92%)	4 (1.42%)	0.858
Ischemic Heart Disease, count (%)	8 (2.2%)	4 (1.42%)	0.669
Using NSAIDs, count (%)	32 (8.79%)	24 (8.54%)	0.977
Using Antiplatelet Therapy, count (%)	13 (3.57%)	9 (3.2%)	0.971
Using PPIs, count (%)	84 (23.08%)	47 (16.73%)	0.059
WBC, mean ± SD, ×10^9^/l	7.07±2.72	7.29±3.26	0.365
Hemoglobin, mean ± SD, g/dl	12.99±1.86	12.8±1.83	0.230
Platelet Count, mean ± SD, ×10^9^/l	306.62±83.03	312.67±83.99	0.377
Creatinine, mean ± SD, mg/dl	0.73±0.21	0.73±0.23	0.959
Total Bilirubin, mean ± SD, mg/dl	0.54±0.32	0.54±0.31	0.811
ALT, mean ± SD, u/l	26.98±16.23	27.21±19.5	0.879
AST, mean ± SD, u/l	21.83±10.46	21.63±10.33	0.822
ALP, mean ± SD, u/l	78.68±23.28	78.75±25.25	0.979
GGT, mean ± SD, u/l	33.41±25.98	39.78±52.44	0.061
Albumin, mean ± SD, g/dl	4.31±5.24	3.95±0.4	0.279
PT, mean ± SD, s	11.9±2.1	11.97±1.32	0.622
PTT, mean ± SD, s	31.27±7.31	31.36±4.44	0.879
INR, mean ± SD	0.94±0.09	1.09±2.28	0.232

## Discussion

There are many reasons behind doing preoperative EGD in bariatric surgery patients. First, the endoscopic findings might lead to choosing a particular type of bariatric surgery type. For example, the findings of complicated GERD, Barrett's esophagus, or severe dysplasia will favor Roux-en-Y gastric bypass (RYGB) over sleeve gastrectomy [30,31]. Secondly, the endoscopic findings might alter the surgical plan. Hiatus hernia, if found, can be repaired concurrently with gastric banding, an intervention that is known to reduce postoperative intractable reflux necessitating reoperation or band removal [32]. Lastly, proceeding with bariatric surgeries without preoperative EGD will risk the surgeons facing surprising incidentalomas that are considered contraindications for surgery. The diagnosis of malignancy in a suspicious endoscopic lesion or the presence of oesophageal varices, for instance, might lead to cancellation of an original bariatric surgery plan [33]. On the other hand, performing a routine preoperative EGD for all bariatric surgery patients is not without any drawbacks. EGDs will add to the medical costs of bariatric procedures. Moreover, bariatric surgeries might get delayed for the EGDs, and delaying such procedures, as done during the coronavirus disease 2019 (COVID-19) crisis, is now known to have medical and psychological impacts on the patients from the continuously increasing weight and the associated depression [34]. 

There are marked local inconsistencies in the Arabian Peninsula about the role of preoperative EGD in bariatric surgery [35]. A total of 65% of 148 International Federation for the Surgery of Obesity and Metabolic Disorders-Middle East and North Africa Chapter (IFSO-MENAC) surgeon members who responded to a survey in 2019 reported that they did not request routine preoperative endoscopy for patients undergoing bariatric surgeries [36]. A study of 1555 patients in Qatar questioned the justifiability of preoperative EGD in asymptomatic patients scheduled for bariatric surgeries in the settings of a low percentage of significant findings that might change the surgical plans (10.5%) and a low rate of upper gastrointestinal cancers in the region [37]. A similar study of 1278 patients in the United Arab Emirates found the opposite, with 63.6% of the patients categorized to have abnormalities that had a direct impact on the surgical procedure and concluded that routine EGD is important for patients planned for bariatric surgery [38]. In Saudi Arabia, multiple smaller studies gave conflicting recommendations with one study advocating for preoperative EGD to be performed only if clinically indicated [39], and the others defending its use as a mandatory investigation for all patients undergoing bariatric surgery [33,40,41]. However, the high number of significant endoscopic and histopathological findings in our relatively larger study also supports the routine use of EGD in all bariatric surgery patients. It is still questionable though whether EGD before RYGB is really needed as the identification of abnormalities such as esophagitis or hiatal hernia by EGD does not usually affect the RYGB plan.

The prevalence of *H. pylori *infections at 53% in our sample of patients with obesity is comparable to the published data of an *H. pylori* prevalence of around 66-73% in obese patients in Saudi Arabia and higher than the known prevalence in non-obese patients, which ranges between 26% and 50% [42,43]. This indicates a possible link between obesity and *H. pylori* infections and could explain at least in part the increased risk of gastric cancer in obese patients [44]. Active surveillance and treatment of *H. pylori *infections in obese patients are important. However, it is unclear whether *H. pylori* should be eradicated before bariatric surgery as the rates of bleeding, leakage, hospital length of stay, and weight loss were comparable between *H. pylori-*positive and *H. pylori-*negative bariatric surgery patients [45,46]. Nevertheless, *H. pylori* is associated with increased marginal ulceration rates in patients undergoing RYGB [45], and postoperative foregut symptoms are more common in *H. pylori-*infected patients [47,48]. Moreover, anti-*H. pylori *antibody seropositivity was found to be associated with de novo gallstone formation [49], a well-known complication of bariatric surgeries [50]. Regrettably, our study failed to find significant predictors of *H. pylori *infection.

Our study has several limitations that should be considered. The first is that the number of included patients would have been bigger if it were not for the COVID-19 crisis and its caused restrictions on elective surgeries. Secondly, the patients were not categorized based on the presence and absence of gastrointestinal symptoms. Therefore, a correlation between the patient's symptoms and the presence of endoscopic findings could not be examined. Similarly, esophagitis was not classified based on the Los Angeles classification grades. Thus, even minimal esophagitis was considered to be a significant endoscopic finding although it might not change the management. In addition, due to the retrospective nature of the research, it was not clear if and to what degree the EGD findings impacted the original surgical plans. Lastly, the long-term postoperative outcomes were not considered.

## Conclusions

The high number of significant endoscopic and histopathological findings in our study supports the routine use of preoperative EGD in all bariatric surgery patients. However, omitting EGD before RYGB in asymptomatic patients is still reasonable as the most frequently found significant findings, esophagitis and hiatus hernia, are less likely to impact the operative plans in RYGB. Similarly, active surveillance and treatment of *H. pylori* infections in obese patients are important but it is not clear whether *H. pylori* eradication should be done before bariatric surgery.
